# Analysis of data with dependent measures in clinical and experimental studies

**DOI:** 10.1590/1677-5449.202201502

**Published:** 2023-05-15

**Authors:** Hélio Amante Miot

**Affiliations:** 1 Universidade Estadual Paulista “Júlio de Mesquita Filho” - UNESP, Faculdade de Medicina de Botucatu - FMB, Departamento de Infectologia, Dermatologia, Diagnóstico por Imagem e Radioterapia, Botucatu, SP, Brasil.

Many different study designs involve the analysis of the same subject (or experimental unit) in different situations or under repeated conditions (Figure 1). This occurs in longitudinal time-dependent assessments (for example, before and after measures, clinical trials, studies of the progress over time of intervention),^1,2^ when measures of different areas of the same subject are assessed (for example, comparisons between adjacent structures: healthy vs. sick and split body interventions),^3,4^ or when measures are obtained from the same organism challenged by different stimuli (for example, response to drugs, temperature, or pain).^5,6^

**Figure 1:**
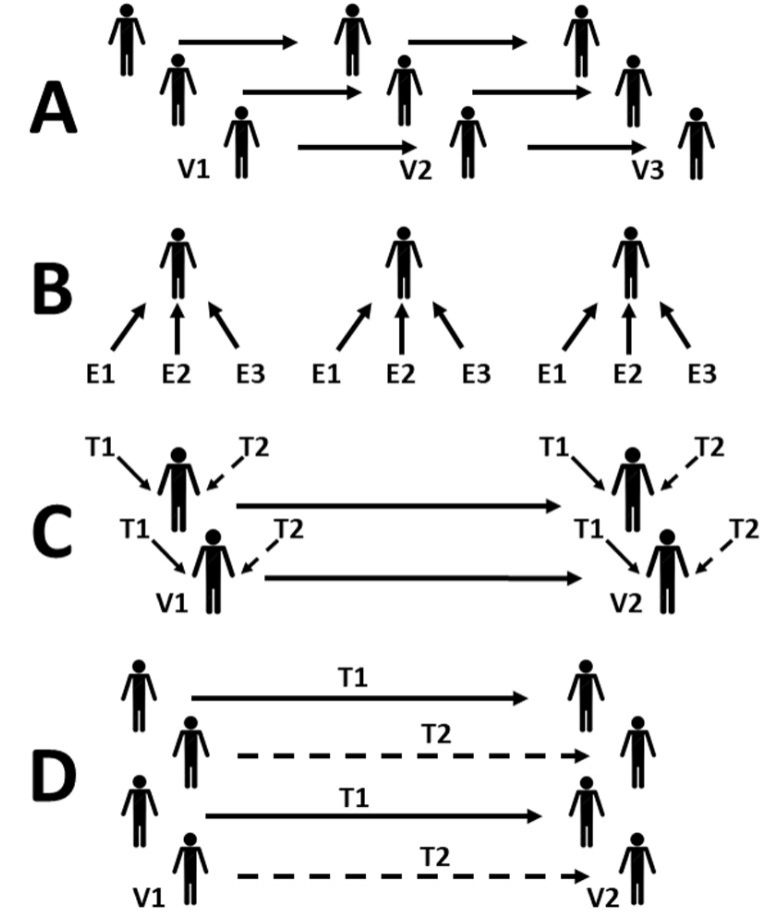
Schematic diagram illustrating study designs in which the measures of variables have some degree of dependence. (A) Longitudinal follow-up over time. The individual measures at visit 1 (V1) should be weighted for analysis of the behavior at subsequent visits (V2 and V3); (B) stimuli assessed in the same organism. Measures provoked by stimuli E1, E2, and E3 should be analyzed according to the organism to which they were administered; (C) simultaneous interventions in the same subject, with longitudinal follow-up. The effect of treatments 1 and 2 (T1 and T2) administered to different sites in the same subject (in a split body trial, for example) should be compared according to the individual response, creating a double dependence: by time (V1 and V2) and by subject; (D) clinical trial. The effects of treatments (T1 and T2) should be analyzed according to the individual effect, over time.

Variables for which there is a link (whether temporal or organic) between different measures generate data that should be analyzed in a dependent manner (paired or correlated), which minimizes the variability between these measures, maximizing the analytical power, and requiring smaller sample sizes for statistical inferences. However, quantitative analysis of dependent data is sensitive to different analytical assumptions, which demands caution when choosing which statistical techniques to employ and when interpreting their results.^7-9^

Didactically, there are four different analytical approaches, based on the concept of “change” in the measures and which guide statistical analysis with different techniques and can lead to different conclusions being drawn from the same set of data. These techniques are (i) identification of change, (ii) comparison of the absolute change in a measure, (iii) comparison of the relative change in a measure, or (iv) conversion of values to a specific outcome.

To illustrate these approaches, Figure 2 shows a hypothetical data distribution of the areas of venous ulcers in a three-armed randomized trial of dressings (A, B, and C) lasting 120 days. Table 1 presents the statistics for these samples according to the four analytical approaches, exploring the nuances of the analysis of data from dependent measures. In this example, the three groups have initial mean values (D0) and final mean values (D120) with no statistical difference between them (p ≥ 0.1), which gives the impression that the behavior of the groups is similar; whereas an individual analysis of the change in their measures (D0-D120) may reveal different conclusions, depending on the analytical assumptions adopted.

**Figure 2:**
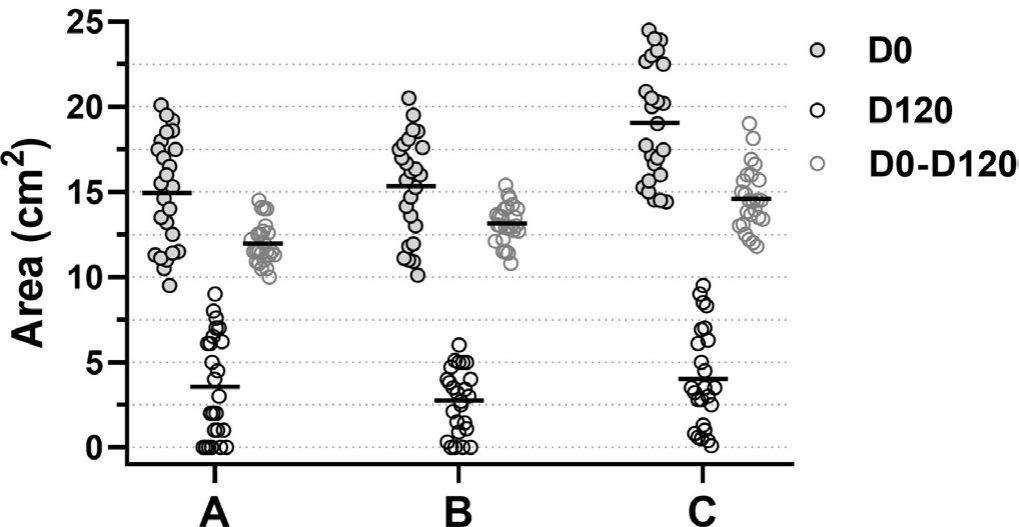
Graphical representation of the area of 75 lower limb venous ulcers treated over 120 days with three different strategies: (A, B, and C) Hypothetical data. D0: baseline area; D120: area after 120 days of treatment; D0-D120: absolute difference in the area from start to end of the clinical trial.

**Table 1:** Description of the areas (cm^2^) of samples of 75 lower limb venous ulcers treated for 120 days with three different strategies, A, B, and C, and the results of analyses using the analytical approaches discussed in the text.

**Descriptive statistics**	**Treatment A**	**Treatment B**	**Treatment C**
D0^*, §^	15.5 (3.0)	15.6 (2.8)	17.1 (2.5)
D120*^,^^§§^	3.5 (3.0)	2.4 (2.0)	2.5 (1.9)
**Evaluation of change**	**Treatment A**	**Treatment B**	**Treatment C**
D0-D120^**^	12.0 (11.5-12.5)	13.1 (12.7-13.6)	14.6 (13.8-15.4)
p-value ^†^	< 0.001	< 0.001	< 0.001
**Absolute difference**	**Treatment A**	**Treatment B**	**Treatment C**
Reduction in area *	12.0 (1.2)	13.1 (1.1)	14.6 (1.8)
	**Treatment A vs. B**	**Treatment A vs. C**	**Treatment B vs. C**
Difference between treatments**	1.2 (0.4-1.9)	2.6 (1.7-3.6)	1.5 (0.6-2.4)
p-value^††^	0.004	<0.001	<0.001
**Relative difference**	**Treatment A**	**Treatment B**	**Treatment C**
Percentage reduction*	79.9 (15.7)	86.0 (10.7)	86.0 (9.4)
	**Treatment A vs. B**	**Treatment A vs. C**	**Treatment B vs. C**
Difference in percentages**^,^^§§§^	6.2 (-1.9-14.2)	5.7 (-2.0-13.4)	0.5 (-6.1-7.1)
Difference adjusted for D0**	1.1 (0.4-1.9)	2.2 (1.3-3.0)	1.0 (0.3-1.7)
p-value^‡^	0.001	< 0.001	0.003
**Outcome: full healing**	**Treatment A**	**Treatment B**	**Treatment C**
n (%)	6 (24%)	4 (16%)	0 (-)
	**Treatment A vs. B**	**Treatment A vs. C**	**Treatment B vs. C**
Difference (%) between groups**	8.0 (-14.1-30.1)	24.0 (3.6-44.4)	16.0 (-0.1-32.4)
p-value^‡‡^	0.477	0.015	0.057

* Mean (standard deviation).

** Mean difference (95% confidence interval).

† *t*-test for paired samples.

†† One-way analysis of variance (ANOVA) (sequential Šidák post hoc).

‡ Generalized linear model - normal distribution (sequential Šidák *post hoc*).

‡‡ Generalized linear model- logit distribution (sequential Šidák *post hoc*).

§ p = 0.054 (one-way ANOVA test).

§§ p = 0.498 (Kruskal-Wallis test).

§§§ p = 0.365 (Kruskal-Wallis test).

In the first analytical approach, the identification of a difference (or change) in status between the situations is based on the hypothesis that the mean difference between measures will be different from zero. This is the condition usually employed in exploratory studies because it is not itself dependent on the dimension of change, but on the probability of identifying a difference between the dependent measures.

For frequency comparisons of paired quantitative measures, the Student’s *t*-test (for paired data) or the Wilcoxon test is used, depending on whether the distribution of the differences between measures is normal.^10^ Comparison of paired ordinal data can be accomplished using the Wilcoxon test and the McNemar test can be used for the comparison of dichotomous data.^11^ As measures of the effect size of this type of analysis, it is usual to present the mean (or median) difference between the pair of measures and its 95% confidence interval.^12^

Simultaneous assessment of more than one pair of quantitative measures of the same subject can be accomplished using repeated measures analysis of variance (ANOVA) or the Friedman test if data normality and sphericity (Mauchly’s test) are not demonstrated. For analysis of multiple dependent ordinal and dichotomous measures, the Friedman test and Cochran’s Q should be used, respectively. Once a difference has been identified using one of these multiple comparison tests, *post hoc* analysis should be used to show which comparisons are responsible for the difference found between times or groups. Several different procedures exist to minimize the error introduced by successive multiple comparisons (for example, Tukey; Bonferroni; Šidák; Scheffé; Ryan-Einot-Gabriel and Welsh Q [REGWQ]; Dunnett; and Games-Howell, etc.), which are based on different theoretical assumptions and which should be chosen with the help of an experienced statistician.^13-16^

In the example shown in Figure 2, all of the treatment groups result in non-zero differences (p < 0.001) in the area of the ulcers (D0 vs. D120), as identified with Student’s *t*-test (for paired data) applied to each treatment separately, showing that all three groups changed their baseline status (Table 1). However, simply identifying a change in the values does not per se provide an adequate dimension of the difference to conduct an analysis between the study groups, preventing detection of the superiority of one treatment over another, albeit the confidence intervals for the differences do offer a certain idea of the intergroup behavior.

At this juncture, the second analytical approach is to only compare the absolute change in the dependent values for different groups (by subtracting before and after, for example), enabling comparisons between measures from longitudinal series with more than two observations or analysis of trials with more than one arm assessed using dependent measures.

This second strategy reduces the complexity of the analysis, and, since only the absolute change in values is compared, the differences in the measures can be compared between study groups using statistical tests for independent samples, such as Student’s *t*-test, the Mann-Whitney test, or Pearson’s chi-square test when making comparisons between two different groups or using ANOVA, the Kruskal-Wallis test, or the chi-square test for more than two groups. However, for longitudinal series with more than two consecutive temporal assessments in a single group, the values for the change with relation to baseline status still maintain a certain dependence on each other and should therefore be analyzed using the techniques described for the first analytical approach.

For the example illustrated in Figure 2 and shown in Table 1, the second analytical approach detects that the absolute change (D0-D120) in the ulcer area was different in each of the three treatment groups (p < 0.001), indicating differences between the interventions. However, this analysis does not consider the importance of the values before the intervention (in this example, the area of the ulcers at D0), the time since disease onset, correct adherence to treatments, the body areas affected, the class of venous insufficiency, or even the patient’s underlying clinical conditions, all of which are elements that could interfere with the absolute change in venous ulcer measurements.^17^ Although exclusively comparing absolute changes in values reduces analytical complexity, it is not routinely employed in clinical research when the experiment is not rigidly controlled or when the phenomenon can be influenced by other factors, such as subjects’ prior status, underlying organic conditions, or environmental stimuli.^9,18^

To deal with this contingency, the third analytical approach consists of analyzing the relative change in the dependent measures. It employs the same tests used to compare the absolute differences but considers the relative change in values. When the data from the example (Figure 2 and Table 1) are compared in terms of percentage change, no differences are found between the treatments (p = 0.365). However, the percentage reductions in these measures may not adequately reflect the baseline status and tend to have nonparametric distributions, imposing a less satisfactory performance on the intergroup comparison of changes.^19^

Indeed, an individual percentage change in a clinical outcome will be larger when the baseline value is smaller. After obesity treatments, patients with larger initial body mass will exhibit larger absolute weight reductions, but smaller percentage reductions than patients with lower weight prior to the intervention.

In this case, returning to the example in Figure 2, when the change in the values of the measurements (D0-D120) is adjusted by their prior values (D0 for example), the reduction in areas can be compared between the three treatments weighted for their relative initial values. If the groups do not differ in terms of their baseline status, as is the case in this example, the use of generalized linear models (analysis of covariance [ANCOVA]), adjusted for the baseline value (or for another type of control), will maximize the analytical power of repeated measures between groups, and this strategy also allows adjustment for other covariates of interest (for example, age or comorbidities) and is widely used for clinical trials and exploratory research.^20-22^ While this approach is sensitive to the detection of changes between groups, it does not necessarily incorporate any considerable clinical significance. In the example illustrated in Figure 2, all of the treatments are different from each other (p < 0.01), and significant differences of the order of 1 cm^2^ can be detected (Table 1), so the clinical relevance of this should be pondered.^12^

The fourth analytical approach to dependent measures considers whether a set outcome has been reached (for example, normalization of blood pressure, 50% flow patency, glycated hemoglobin levels of < 7%, complete healing of the ulcer, or absence of claudication after walking four blocks). From the pragmatic point of view, dichotomous outcomes (known as “hard” outcomes) have a highly understandable meaning that can be transposed to clinical practice and are frequently used as the primary endpoints of clinical trials. Dichotomous outcomes are analyzed with techniques for the comparison of proportions between groups, represented by the percentage of events and its 95% confidence interval.^23^

Analysis of dichotomous outcomes as the parameter of change in the analysis of data from dependent measures offers less statistical power and requires larger samples than the analytical techniques used in the earlier situations and is fundamentally dependent on the prior condition of the measures in the subjects. In the example shown in Figure 2 (Table 1), although treatment C provoked a larger numerical reduction in ulcer area, this was also the intervention that least induced full healing, which could be because of the prior status of the ulcers themselves, with subjects having greater initial ulcer area, but could also be because of clinical conditions that interfere with healing. This is relevant in the comparison of clinical trials or in the evaluation of the results of meta-analyses, because the participants’ baseline conditions (for example, age, weight, comorbidities, disease severity, and metabolic status) may differ between studies, interfering in the achievement of outcomes, irrespective of the treatment analyzed.^24,25^ Moreover, although logistic models can be adjusted for other covariates, this type of correction is not usually employed in the analysis of clinical trials.

Since all four analytical approaches are absolutely correct and justifiable, it should be remembered that each may lead to different conclusions with respect to the same study. It is therefore the researcher’s prerogative to define, a priori, which approach will be taken, while the analytical techniques, the objectives, and the results obtained are all conditioned by the strategy employed.^26^ Moreover, when describing data from repeated measures, care should be taken to present the results in line with the analytical objectives required and the discussion of the results should cover the limitations of using one or another of the possible approaches.

As the structure of the study data acquires a certain degree of complexity, such as several repetitions, comparison of repetitions between groups, dependence in more than one condition, different baseline status between groups, inadequate sphericity, the need to weight results for the behavior of other covariates, covariance structures between less common measures, or where longitudinal follow-up times are not set for all observations, analytical modeling should tend towards generalized estimating equations (GEE) or generalized linear mixed-effects models. These techniques can be adapted for the analysis of unimodal quantitative variables (with normal or asymmetrical distributions), count type variables, and ordinal, multinomial, or dichotomous qualitative variables, making the analysis more versatile and a better fit to the data.^27-31^

Along the same lines, analytical designs exist that demand simultaneous study of different variables from the same subject, creating a structure of dependence within the individual, as is the case of quality of life scales that assess more than one dimension (for example, the Venous Insufficiency Epidemiological and Economic Study - Quality of Life/Symptom [VEINES-QoL/Sym], Skindex-17), different sets of symptoms, or different serum markers secreted after a single stimulus.^32-34^ Quantitative analysis of groups using simultaneous analysis of more than one dependent variable demands the use of methods known as multivariate, such as profile analysis, multivariate analysis of variance (MANOVA), permutational multivariate analysis of variance (PERMANOVA), canonic correlation, or Log-linear models (multivariate),^23,35-37^ but their complexity demands supervision by a statistics professional with experience in this type of modeling.

Finally, while study designs involving data with dependent measures increase the statistical power of the analysis, it is necessary to present a highly detailed description of the analytical objectives and the statistical techniques employed, since they have direct implications for sample sizing and the type of results provided by the study.

## References

[1] Pereira LA, Luz FB, Carneiro C, Xavier ALR, Kanaan S, Miot HA (2019). Evaluation of vitamin D plasma levels after mild exposure to the sun with photoprotection. An Bras Dermatol.

[2] Saliba-Júnior OA, Rollo HA, Saliba O, Sobreira ML (2022). Positive perception and efficacy of compression stockings for prevention of lower limb edema in pregnant women. J Vasc Bras.

[3] Miola AC, Ferreira ER, Lima TRR, Schmitt JV, Abbade LPF, Miot HA (2018). Effectiveness and safety of 0.5% colchicine cream vs. photodynamic therapy with methyl aminolaevulinate in the treatment of actinic keratosis and skin field cancerization of the forearms: a randomized controlled trial. Br J Dermatol.

[4] Espósito ACC, Brianezi G, Souza NP, Miot LDB, Miot HA (2020). Exploratory study of epidermis, basement membrane zone, upper dermis alterations and wnt pathway activation in melasma compared to adjacent and retroauricular skin. Ann Dermatol.

[5] Maciel-Guerra H, Penha MA, Jorge MFS (2018). Suppression of wheal and flare in histamine test by the main H1 antihistamines commercialized in Brazil. An Bras Dermatol.

[6] Kitahara LBW, Silva VPD, Peres G, Miot HA, Schmitt JV (2021). Efficacy of different concentrations of lidocaine and association of vasoconstrictor in local infiltration anesthesia in adults. An Bras Dermatol.

[7] Lord FM (1967). A paradox in the interpretation of group comparisons. Psychol Bull.

[8] Wainer H, Messick S (2012). Principals of modern psychological measurement: a festschrift for Frederic M. Lord.

[9] Senn S (2006). Change from baseline and analysis of covariance revisited. Stat Med.

[10] Miot HA (2017). Assessing normality of data in clinical and experimental trials. J Vasc Bras.

[11] Miot HA (2020). Analysis of ordinal data in clinical and experimental studies. J Vasc Bras.

[12] Miola AC, Miot HA (2021). P-value and effect-size in clinical and experimental studies. J Vasc Bras.

[13] Elliott HL (1996). Post hoc analysis: use and dangers in perspective. J Hypertens Suppl.

[14] Ruxton GD, Beauchamp G (2008). Time for some a priori thinking about post hoc testing. Behav Ecol.

[15] Lee S, Lee DK (2018). What is the proper way to apply the multiple comparison test?. Korean J Anesthesiol.

[16] Norman GR, Streiner DL (2015). Biostatistics: the bare essentials.

[17] Scotton MF, Miot HA, Abbade LP (2014). Factors that influence healing of chronic venous leg ulcers: a retrospective cohort. An Bras Dermatol.

[18] Norman GR (1989). Issues in the use of change scores in randomized trials. J Clin Epidemiol.

[19] Vickers AJ (2001). The use of percentage change from baseline as an outcome in a controlled trial is statistically inefficient: a simulation study. BMC Med Res Methodol.

[20] O'Connell NS, Dai L, Jiang Y (2017). Methods for analysis of pre-post data in clinical research: a comparison of five common methods. J Biom Biostat.

[21] Austin PC, Manca A, Zwarenstein M, Juurlink DN, Stanbrook MB (2010). A substantial and confusing variation exists in handling of baseline covariates in randomized controlled trials: a review of trials published in leading medical journals. J Clin Epidemiol.

[22] van Breukelen GJ (2013). ANCOVA versus CHANGE from baseline in nonrandomized studies: the difference. Multivariate Behav Res.

[23] Miola AC, Miot HA (2022). Comparing categorical variables in clinical and experimental studies. J Vasc Bras.

[24] Eysenck HJ (1994). Meta-analysis and its problems. BMJ.

[25] El Dib R (2022). How to interpret a meta-analysis?. J Vasc Bras.

[26] Twisk J, Proper K (2004). Evaluation of the results of a randomized controlled trial: how to define changes between baseline and follow-up. J Clin Epidemiol.

[27] Albert PS (1999). Longitudinal data analysis (repeated measures) in clinical trials. Stat Med.

[28] Bandyopadhyay S, Ganguli B, Chatterjee A (2011). A review of multivariate longitudinal data analysis. Stat Methods Med Res.

[29] Fitzmaurice GM, Ravichandran C (2008). A primer in longitudinal data analysis. Circulation.

[30] Twisk JW (2013). Applied longitudinal data analysis for epidemiology: a practical guide.

[31] Araújo IC, Defune E, Abbade LP (2017). Fibrin gel versus papain gel in the healing of chronic venous ulcers: a double-blind randomized controlled trial. Phlebology.

[32] Ribeiro-Samora GA, Carvalho MLV, Moura RMF, Pereira DAG (2019). Limitations of VEINES QOL/SYM for discriminating chronic venous insufficiency severity. J Vasc Bras.

[33] Almeida IL, Figueiredo PHS, Silva WT (2022). Reliability and validity of specific quality of life assessment questionnaires related to chronic venous insufficiency: a systematic review. J Vasc Bras.

[34] Jorge MFS, Mourao IB, Pollo CF, Sousa TD, Meneguin S, Miot HA (2021). Validation of the Skindex-17 quality of life assessment instrument for a Brazilian population. An Bras Dermatol.

[35] Davison ML, Kim S-K, Close C (2009). Factor analytic modeling of within person variation in score profiles. Multivariate Behav Res.

[36] Liu Y, Carmer R, Zhang G (2015). Statistical analysis of zebrafish locomotor response. PLoS One.

[37] Morrow GR, Black PM, Dudgeon DJ (1991). Advances in data assessment. Application to the etiology of nausea reported during chemotherapy, concerns about significance testing, and opportunities in clinical trials. Cancer.

