# Supplementary material for: Analysis of data with dependent measures in clinical and experimental studies
Source: J Vasc Bras. 2023 May 15;22:e20220150. doi: 10.1590/1677-5449.202201502 (PMC10275643; doi:10.1590/1677-5449.202201502)
Supplement: Translation

# Translation: Análise de dados com medidas dependentes em estudos clínicos e experimentais

**DOI:** 10.1590/1677-5449.202201501

**Authors:** Hélio Amante Miot

**Affiliations:** 1 Universidade Estadual Paulista “Júlio de Mesquita Filho” - UNESP, Faculdade de Medicina de Botucatu - FMB, Departamento de Infectologia, Dermatologia, Diagnóstico por Imagem e Radioterapia, Botucatu, SP, Brasil.

Diversos desenhos de pesquisa analisam o mesmo sujeito (ou unidade experimental) em diferentes situações ou sob condições repetidas (Figura 1). Isso ocorre nas avaliações longitudinais dependentes do tempo (por exemplo, medidas antes e depois, ensaios clínicos, evolução temporal de uma intervenção)^1,2^ , nas medições em topografias diferentes do mesmo sujeito (por exemplo, comparação entre estruturas adjacentes: saudáveis *versus* doentes, intervenções *split body*)^3,4^ ou nas medidas oriundas do mesmo organismo desafiado por diferentes estímulos (por exemplo, resposta a drogas, temperatura ou dor)^5,6^ .

**Figura 1:** Representação esquemática de desenhos de estudos em que as medidas das variáveis apresentam alguma dependência. (A) Seguimento longitudinal em função do tempo. As medidas individuais da visita 1 (V1) devem ser ponderadas para a análise do comportamento das visitas subsequentes (V2 e V3); (B) estímulos avaliados no mesmo organismo. As medidas decorrentes dos estímulos E1, E2 e E3 devem ser analisadas segundo o organismo em que foram implementados; (C) intervenções simultâneas no mesmo sujeito, com seguimento longitudinal. O efeito dos tratamentos 1 e 2 (T1 e T2) implementados em topografias diferentes do mesmo sujeito (por exemplo, *split body*) devem ser comparados segundo a resposta individual, gerando uma dupla dependência: segundo o tempo (V1 e V2) e o indivíduo; (D) ensaio clínico. Os efeitos dos tratamentos (T1 e T2) devem ser analisados segundo o efeito individual, em função do tempo.

Variáveis que mantêm certo vínculo (temporal ou orgânico) entre as medidas de seus sujeitos geram dados que devem ser analisados de maneira dependente (emparelhada ou correlata), o que minimiza a variabilidade entre tais medidas, maximizando o poder da análise e demandando menor tamanho amostral para a inferência estatística. Contudo, a análise quantitativa de dados dependentes é sensível a diferentes pressupostos analíticos que exigem cautela quanto à escolha das técnicas estatísticas empregadas e à interpretação de seus resultados^7-9^ .

Didaticamente, há quatro abordagens analíticas diferentes, baseadas no conceito da “mudança” entre as medidas, que direcionam a análise estatística segundo técnicas diferentes e que podem gerar conclusões distintas a partir do mesmo conjunto de dados. São elas (i) a identificação de mudança, (ii) a comparação da mudança absoluta das medidas, (iii) a comparação da mudança relativa das medidas e (iv) a mudança dos valores para um desfecho específico.

Para exemplificar essas abordagens, a Figura 2 apresenta a distribuição de dados hipotéticos da área de úlceras venosas submetidas a um ensaio randomizado de três braços com curativos (A, B e C) por 120 dias. A Tabela 1 apresenta as estatísticas referentes a essas amostras segundo as quatro abordagens analíticas e explora as nuances da análise de dados com medidas dependentes. Nesse exemplo, os três grupos apresentam tanto os valores médios iniciais (D0) quanto os finais (D120) sem diferença estatística entre si (p ≥ 0,1), induzindo a percepção de semelhança no comportamento entre os grupos; porém, a análise individual das mudanças entre suas medidas (D0-D120) pode desvelar conclusões diferentes, dependendo do pressuposto analítico utilizado.

**Figura 2:** Representação gráfica das áreas de 75 úlceras venosas dos membros inferiores tratadas por 120 dias com três estratégias diferentes: (A, B e C) Dados hipotéticos. D0: área basal; D120: área após 120 dias de tratamento; D0-D120: diferença absoluta entre as áreas do início e fim do ensaio clínico.

**Tabela 1:** Descrição das áreas (cm^2^) das amostras de 75 úlceras venosas dos membros inferiores tratadas por 120 dias com três estratégias diferentes, A, B e C, e resultados da análise segundo as abordagens analíticas discutidas no texto.

**Estatística descritiva**	**Tratamento A**	**Tratamento B**	**Tratamento C**
D0^*,^^§^	15,5 (3,0)	15,6 (2,8)	17,1 (2,5)
D120*^,^^§§^	3,5 (3,0)	2,4 (2,0)	2,5 (1,9)
**Avaliação da mudança**	**Tratamento A**	**Tratamento B**	**Tratamento C**
D0-D120^**^	12,0 (11,5-12,5)	13,1 (12,7-13,6)	14,6 (13,8-15,4)
Valor de p^†^	<0,001	<0,001	<0,001
**Diferença absoluta**	**Tratamento A**	**Tratamento B**	**Tratamento C**
Redução da área*	12,0 (1,2)	13,1 (1,1)	14,6 (1,8)
	**Tratamento A vs. B**	**Tratamento A vs. C**	**Tratamento B vs. C**
Diferença entre os tratamentos**	1,2 (0,4-1,9)	2,6 (1,7-3,6)	1,5 (0,6-2,4)
Valor de p^††^	0,004	<0,001	<0,001
**Diferença relativa**	**Tratamento A**	**Tratamento B**	**Tratamento C**
Percentual de redução*	79,9 (15,7)	86,0 (10,7)	86,0 (9,4)
	**Tratamento A vs. B**	**Tratamento A vs. C**	**Tratamento B vs. C**
Diferença dos valores percentuais**^,^^§§§^	6,2 (-1,9-14,2)	5,7 (-2,0-13,4)	0,5 (-6,1-7,1)
Diferença ajustada pelo D0**	1,1 (0,4-1,9)	2,2 (1,3-3,0)	1,0 (0,3-1,7)
Valor de p^‡^	0,001	<0,001	0,003
**Desfecho: cicatrização completa**	**Tratamento A**	**Tratamento B**	**Tratamento C**
n (%)	6 (24%)	4 (16%)	0 (-)
	**Tratamento A vs. B**	**Tratamento A vs. C**	**Tratamento B vs. C**
Diferença (%) entre os grupos**	8,0 (-14,1-30,1)	24,0 (3,6-44,4)	16,0 (-0,1-32,4)
Valor de p^‡‡^	0,477	0,015	0,057

* Média (desvio-padrão).

** Diferença média (intervalo de confiança de 95%). ^†^Teste *t* de amostras emparelhadas.

†† Análise de variância (ANOVA) de uma via (*post hoc* Šidák sequencial).

‡ Modelo linear generalizado - distribuição normal (*post hoc* Šidák sequencial).

‡‡ Modelo linear generalizado - distribuição *logit* (*post hoc* Šidák sequencial).

§ p = 0,054 (teste ANOVA de uma via).

§§ p = 0,498 (teste de Kruskal-Wallis).

§§§ p = 0,365 (teste de Kruskal-Wallis).

Na primeira abordagem analítica, a verificação de diferença (ou de mudança) no *status* entre as situações se baseia na hipótese de que a média da diferença entre as medidas resulta um valor diferente de zero. Essa é a condição usualmente empregada em estudos exploratórios, porque não depende propriamente da dimensão da mudança, mas da probabilidade de se identificar alguma diferença entre as medidas dependentes.

Para a comparação frequentista entre pares de medidas quantitativas, são empregados os testes *t* de Student (para dados emparelhados) ou de Wilcoxon, dependendo da normalidade da distribuição das diferenças entre as medidas^10^ . A comparação entre pares de dados ordinais pode ser realizada pelo teste de Wilcoxon, e a comparação entre dados dicotômicos, pelo teste de McNemar^11^ . Como medida de efeito desse tipo de análise, é usual que se represente a diferença média (ou mediana) do par de medidas e o seu intervalo de confiança de 95%^12^ .

Já a avaliação simultânea de mais de um par de medidas quantitativas do mesmo sujeito pode ser realizada pelo teste de análise de variância (ANOVA) de medidas repetidas ou pelo teste de Friedman, se a normalidade e a esfericidade (teste de Mauchly) dos dados não forem identificadas. Para a análise de múltiplas medidas ordinais e dicotômicas dependentes, devem ser empregados os testes de Friedman e Q de Cochran, respectivamente. Uma vez identificada a diferença em um desses testes de comparações múltiplas, é indicada a análise *post hoc* para evidenciar quais comparações são responsáveis pela diferença encontrada entre os tempos ou os grupos. Há diferentes procedimentos que visam minimizar o erro decorrente das múltiplas comparações sucessivas (por exemplo, Tukey, Bonferroni, Šidák, Scheffé, Ryan-Einot-Gabriel- Welsh Q [REGWQ], Dunnett, Games-Howell etc.) que seguem diferentes pressupostos teóricos, e tal escolha pode demandar a orientação de um estatístico experiente^13-16^ .

No exemplo da Figura 2, todos os grupos de tratamento resultaram em diferenças das áreas das úlceras (D0 *versus* D120) diferentes de zero (p < 0,001), identificadas pelo teste *t* de Student (para dados emparelhados) aplicado a cada tratamento de forma separada, reconhecendo que todos os três grupos modificaram seu *status* inicial (Tabela 1). Entretanto, a simples verificação de mudança entre os valores *per se* não fornece uma dimensão adequada dessa diferença para a análise entre os grupos do mesmo estudo, impedindo a percepção de superioridade de um tratamento sobre o outro, apesar de os intervalos de confiança das diferenças permitirem certa percepção do comportamento entre os grupos.

Nesse ínterim, a segunda abordagem analítica compara unicamente a mudança absoluta (por exemplo, subtração de antes e depois) entre os valores dependentes de diferentes grupos, permitindo a sua comparação entre medidas de séries longitudinais com mais de duas observações ou, ainda, a análise de ensaios com mais de um grupo avaliado com medidas dependentes.

Essa segunda estratégia reduz a complexidade da análise, e, já que se busca comparar exclusivamente a mudança absoluta dos valores, podem-se comparar as diferenças das medidas entre os grupos do estudo por testes estatísticos para amostras independentes, como o teste *t* de Student, o teste de Mann-Whitney ou o teste do qui-quadrado de Pearson, no caso de comparações entre dois grupos diferentes, ou a ANOVA, o teste de Kruskal-Wallis ou o teste do qui-quadrado para mais de dois grupos. Entretanto, no caso de séries longitudinais, com mais de duas avaliações temporais consecutivas em um grupo, os valores de mudança em relação ao *status* inicial ainda mantêm certa dependência entre si e devem ser avaliados pelas técnicas descritas na primeira abordagem analítica.

No exemplo da Figura 2 e Tabela 1, de acordo com a segunda abordagem analítica, identificou-se que a variação absoluta (D0-D120) das áreas das úlceras foi diferente entre todos os grupos de tratamento (p < 0,001), indicando diferença entre as intervenções. Entretanto, essa análise não considera a importância dos valores prévios à intervenção (por exemplo, área da úlcera no D0), o tempo de evolução da doença, a correta aderência aos tratamentos, as topografias afetadas, a classe de insuficiência venosa e tampouco as condições clínicas subjacentes dos pacientes, já que todos esses elementos podem interferir na variação absoluta das medidas de úlceras venosas^17^ . Apesar de reduzir a complexidade da análise, a comparação exclusiva da variação absoluta dos valores não é empregada rotineiramente em pesquisa clínica quando o experimento não for rigidamente controlado ou o fenômeno puder ser influenciado por outros fatores, como *status* prévio dos sujeitos, condições orgânicas subjacentes ou estímulos ambientais^9,18^ .

Para lidar com essa contingência, a terceira abordagem analítica se refere à avaliação da mudança relativa das medidas dependentes. Ela utiliza os mesmos testes utilizados na comparação das diferenças absolutas, mas considera a variação relativa dos valores. Quando os dados do exemplo (Figura 2 e Tabela 1) são comparados pela mudança percentual, não são evidenciadas diferenças entre os tratamentos (p = 0,365). Na verdade, as reduções percentuais das medidas podem não refletir adequadamente o *status* inicial e costumam assumir distribuições não paramétricas, infligindo um desempenho menos satisfatório na comparação da mudança entre os grupos^19^ .

De fato, o percentual de mudança individual de um desfecho clínico é maior quando o valor basal é menor. Em tratamentos de obesidade, pacientes com maior massa corporal podem apresentar maior redução absoluta do peso, mas menor redução percentual quando comparados com pacientes de menor *status* ponderal anterior à intervenção.

Nesse caso, voltando ao exemplo da Figura 2, quando a variação dos valores das medidas (D0-D120) é ajustada pelos valores prévios (por exemplo, D0), permite-se a comparação das reduções das áreas entre os três tratamentos, ponderados relativamente pelo valor inicial. Se os grupos não diferirem entre si quanto ao *status* inicial, como ocorreu nesse exemplo, o emprego de modelos lineares generalizados (análise de covariância [ANCOVA]), ajustados pelo valor inicial (ou por outro tipo de controle), maximiza o poder analítico de medidas repetidas entre grupos, além de essa estratégia permitir o ajuste por outras covariáveis de interesse (por exemplo, idade, comorbidades), sendo uma estratégia largamente utilizada em ensaios clínicos e pesquisas exploratórias^20-22^ . Embora essa abordagem seja sensível à percepção de mudança entre os grupos, ela não incorpora necessariamente um significado clínico tão expressivo. No exemplo da Figura 2, todos os tratamentos diferiram entre si (p < 0,01), permitindo constatar diferenças significativas da ordem de 1 cm^2^ (Tabela 1), o que deve ser ponderado quanto à relevância clínica^12^ .

A quarta abordagem analítica para medidas dependentes considera um desfecho fixo a ser atingido (por exemplo, normalização da pressão arterial, 50% de perviedade de fluxo, níveis de hemoglobina glicada < 7%, cicatrização completa da úlcera, ausência de claudicação após quatro quadras de caminhada). Do ponto de vista pragmático, os desfechos dicotômicos (chamados “duros”) têm seu significado bastante inteligível e transponível para a prática clínica, sendo frequentemente utilizados como os desfechos primários de ensaios clínicos. Desfechos dicotômicos são analisados por técnicas de comparação de proporções entre grupos, representados pelo percentual de eventos e seu intervalo de confiança de 95%^23^ .

A análise de desfechos dicotômicos, como parâmetro de mudança na análise de dados com medidas dependentes, apresenta menor poder estatístico, demandando maiores amostras que as técnicas analíticas usadas nas situações anteriores, e depende fundamentalmente da condição prévia das medidas dos sujeitos. No exemplo da Figura 2 (Tabela 1), apesar de o tratamento C promover uma maior redução numérica da área das úlceras, essa foi a intervenção que menos induziu cicatrização completa, o que pode se dever à própria condição prévia de úlceras com sujeitos apresentando maior área inicial, mas também a condições clínicas que interferissem na cicatrização. Isso é relevante na comparação de ensaios clínicos ou na avaliação do resultado de metanálises, porque as condições basais dos participantes (por exemplo, idade, peso, comorbidades, gravidade da doença, *status* metabólico) podem ser diferentes entre os estudos, interferindo na obtenção de desfechos, independentemente do tratamento avaliado^24,25^ . Ainda, apesar de modelos logísticos poderem ser ajustados para outras covariáveis, essa não é uma correção usualmente empregada na análise de ensaios clínicos.

Uma vez que todas as quatro abordagens analíticas são absolutamente corretas e justificáveis, deve-se ter ciência que elas podem levar a conclusões diferentes para o mesmo estudo. Portanto, é prerrogativa do pesquisador a definição dessa abordagem *a priori*, já que as técnicas de análise, os objetivos e as respostas obtidas são condicionadas pela estratégia utilizada^26^ . Enquanto isso, na etapa de descrição de dados oriundos de medidas repetidas, deve-se atentar à apresentação dos resultados segundo os objetivos analíticos demandados, sendo que a discussão dos resultados deve incluir as limitações de se considerar uma ou outra abordagem.

À medida que a estrutura dos dados do estudo adquire certa complexidade, como várias repetições, comparação de repetições entre grupos, dependência em mais de uma condição, *status* basal diferente entre grupos, esfericidade inadequada, necessidade de ponderação dos resultados pelo comportamento de outras covariáveis, estruturas de covariância entre as medidas menos usuais ou quando os tempos de seguimento longitudinal não forem fixos para todas as observações, a modelagem da análise deve ser conduzida por equações de estimativas generalizadas (GEE) ou modelos lineares generalizados de efeitos mistos. Essas técnicas podem ser adaptadas para analisar variáveis quantitativas unimodais (com distribuição normal ou assimétrica), variáveis tipo contagem e variáveis qualitativas ordinais, multinominais ou dicotômicas, o que torna a análise mais versátil e melhor ajustada aos dados^27-31^ .

Da mesma forma, há desenhos analíticos que demandam o estudo simultâneo de diferentes variáveis do mesmo sujeito, gerando uma estrutura de dependência dentro do indivíduo, como ocorre em escalas de qualidade de vida que avaliam mais de uma dimensão (por exemplo, Venous Insufficiency Epidemiological and Economic Study - Quality of Life/Symptom [VEINES-QoL/Sym], Skindex-17), diferentes conjuntos de sintomas ou diferentes marcadores séricos secretados após um mesmo estímulo^32-34^ . A avaliação quantitativa de grupos segundo a análise simultânea de mais de uma variável dependente exige o emprego de métodos chamados multivariados, como análise de perfil, análise multivariada de variância (MANOVA), análise multivariada de variância com permutação (PERMANOVA), correlação canônica ou modelos Log-lineares (multivariados)^23,35-37^ , entretanto sua complexidade requer a supervisão de um profissional estatístico experiente com esses tipos de modelagem.

Finalmente, ao passo que desenhos de estudos que envolvam dados com medidas dependentes aumentam o poder estatístico da análise, é necessária uma descrição minuciosa dos objetivos analíticos e as técnicas estatísticas empregadas, uma vez que isso implica diretamente no dimensionamento amostral e no tipo de resposta fornecida pelo estudo.
